# Supplementary material for: Tannylated Calcium Carbonate Materials with Antacid, Anti-Inflammatory, and Antioxidant Effects
Source: Int J Mol Sci. 2021 Apr 28;22(9):4614. doi: 10.3390/ijms22094614 (PMC8124724; doi:10.3390/ijms22094614)
Supplement: Supplementary file 1 [file ijms-22-04614-s001.zip › ijms-1183077-supplementary.pdf]

Supplementary Materials

Tannylated Calcium Carbonate Materials with Antacid, Anti-inflammatory, and Antioxidant Effects

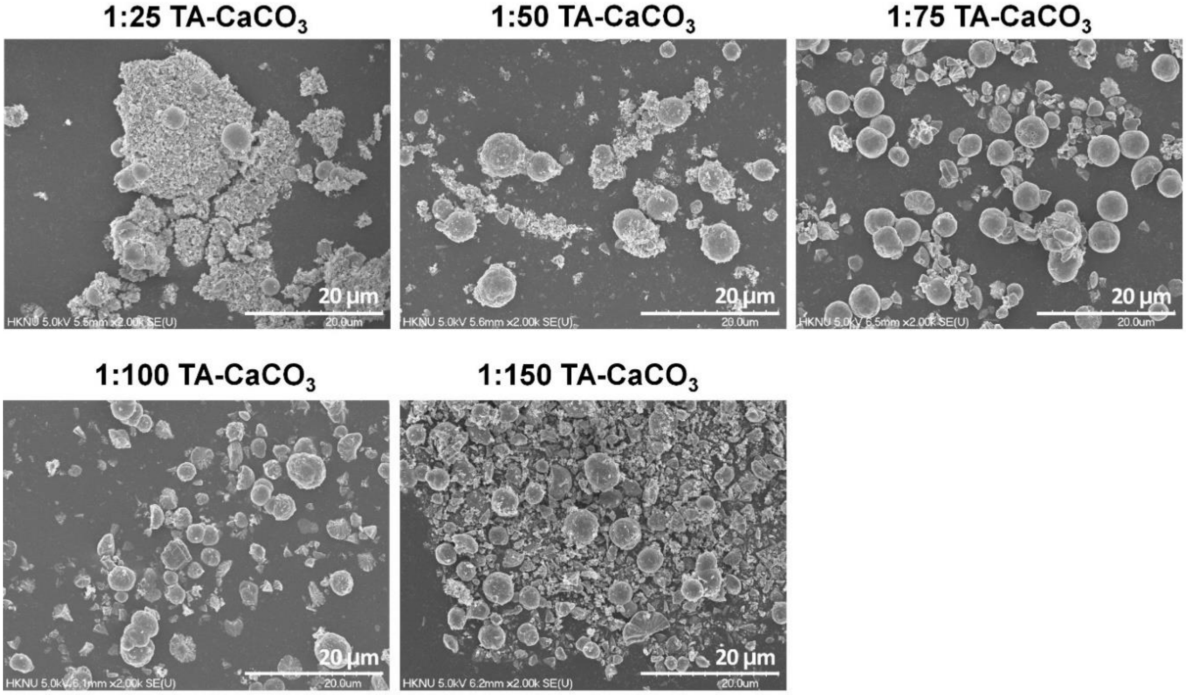

Figure S1. SEM images of the TA-$CaCO_3$ materials synthesized at different molar ratios of TA and $CaCl_2$/Na_2CO_3.

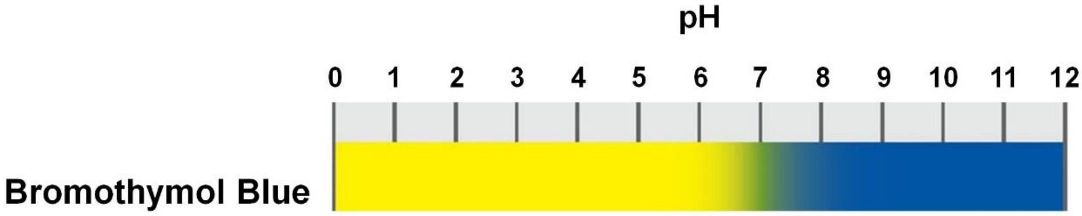

Figure S2. Color changes of BTB at different pH values.

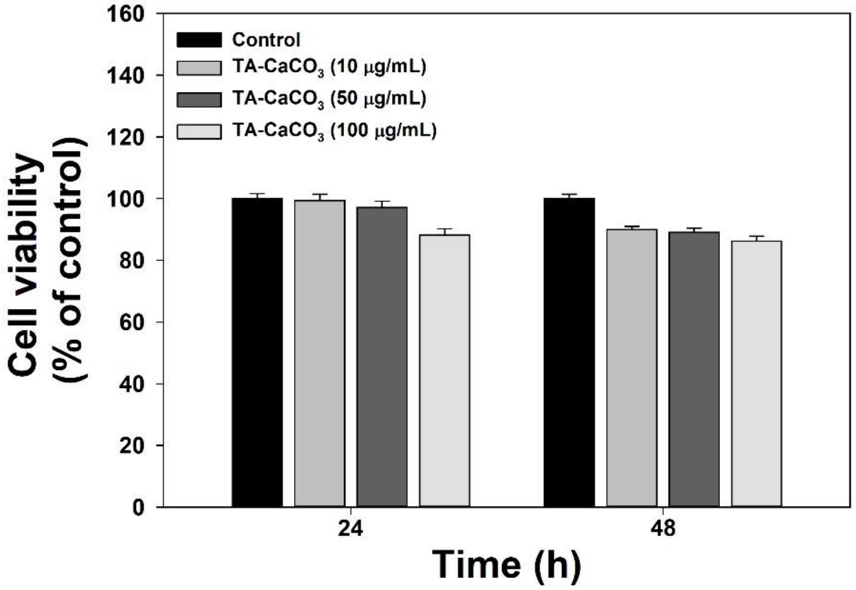

Figure S3. In vitro cytotoxicity of 1:75 TA-$CaCO_3$ against chondrocytes.

Table S1. RT-PCR primer sequences for genes related to pro-inflammatory factors.

Genes	Primer (forward) 5′→ 3′	Primer (reverse)
COX-2	TAT CAG GTC ATC GGT GGA GA	CGA AGC CAG ATG GTA GCA TA
IL-1β	CCA CCT CCA GGG ACA GGA TA	AAC ACG CAG GAC AGG TAC AG
MMP-3	ACC TGT CCC TCC AGA ACC TG	AAC TTC ATA TGC GGC ATC CA
MMP-13	GGT CCT TGG AGT GGT CAA GA	AAG GAG CAT GGC GAC TTC TA
IL-6	GCC TTC GGT CCA GTT GCC	CTC AGG GCT GAG ATG CCG
TNF-α	GAC CCT CAC ACT CAG ATC ATC TTC T	TGC TAC GAC GTG GGC TAC G
